# Supplementary material for: A Selenophene-Incorporated Metal–Organic Framework for Enhanced CO2 Uptake and Adsorption Selectivity
Source: Molecules. 2020 Sep 24;25(19):4396. doi: 10.3390/molecules25194396 (PMC7582736; doi:10.3390/molecules25194396)

checkCIF/PLATON report

Structure factors have been supplied for datablock(s) ZnSedcdabco

THIS REPORT IS FOR GUIDANCE ONLY. IF USED AS PART OF A REVIEW PROCEDURE FOR PUBLICATION, IT SHOULD NOT REPLACE THE EXPERTISE OF AN EXPERIENCED CRYSTALLOGRAPHIC REFEREE.

No syntax errors found. CIF dictionary Interpreting this report

Datablock: ZnSedcdabco

Bond precision: C-C = 0.0042 A Wavelength=0.71073

Cell:	a=20.59224(17)	b=20.59224(17)	c=19.1260(2)
	alpha=90	beta=90	gamma=90
Temperature:	130 K		

	Calculated	Reported
Volume	8110.20(16)	8110.20(16)
Space group	P -4 21 c	P -4 21 c
Hall group	P -4 2n	P -4 2n
Moiety formula	C18 H16 N2 O8 Se2 Zn2, 4(C3 H7 N O)	?
Sum formula	C30 H44 N6 O12 Se2 Zn2	C30 H44 N6 O12 Se2 Zn2
Mr	969.41	969.37
Dx,g cm-3	1.588	1.588
Z	8	8
Mu (mm-1)	3.040	3.040
F000	3920.0	3920.0
F000'	3924.67	
h,k,lmax	27,27,25	27,25,25
Nref	10069[5455]	8741
Tmin,Tmax	0.403,0.544	0.885,1.000
Tmin'	0.322	

Correction method= # Reported T Limits: Tmin=0.885 Tmax=1.000
AbsCorr = MULTI-SCAN

Data completeness= 1.60/0.87 Theta(max)= 28.279

R(reflections)= 0.0223(8293) wR2(reflections)= 0.0502(8741)

S = 1.044 Npar= 497

The following ALERTS were generated. Each ALERT has the format
test-name_ALERT_alert-type_alert-level.
Click on the hyperlinks for more details of the test.

Alert level C

```
STRVA01_ALERT_4_C           Flack test results are ambiguous.
           From the CIF: _refine_ls_abs_structure_Flack    0.491
           From the CIF: _refine_ls_abs_structure_Flack_su    0.008
PLAT242_ALERT_2_C Low    'MainMol' Ueq as Compared to Neighbors of        N2 Check
PLAT244_ALERT_4_C Low    'Solvent' Ueq as Compared to Neighbors of       N4D Check
PLAT244_ALERT_4_C Low    'Solvent' Ueq as Compared to Neighbors of       N3D Check
PLAT250_ALERT_2_C Large U3/U1 Ratio for Average U(i,j) Tensor ....       2.2 Note
PLAT601_ALERT_2_C Unit Cell Contains Solvent Accessible VOIDS of .        34 Ang**3
```

Alert level G

```
PLAT002_ALERT_2_G Number of Distance or Angle Restraints on AtSite          5 Note
PLAT003_ALERT_2_G Number of Uiso or Uij Restrained non-H Atoms ...          5 Report
PLAT004_ALERT_5_G Polymeric Structure Found with Maximum Dimension          3 Info
PLAT111_ALERT_2_G ADDSYM Detects New (Pseudo) Centre of Symmetry .         86 %Fit
PLAT113_ALERT_2_G ADDSYM Suggests Possible Pseudo/New  Space Group    P42/nmc Check
PLAT176_ALERT_4_G The CIF-Embedded .res File Contains SADI Records          2 Report
PLAT178_ALERT_4_G The CIF-Embedded .res File Contains SIMU Records          1 Report
PLAT302_ALERT_4_G Anion/Solvent/Minor-Residue Disorder (Resd  2  )        40% Note
PLAT720_ALERT_4_G Number of Unusual/Non-Standard Labels ..........          6 Note
PLAT794_ALERT_5_G Tentative Bond Valency for Zn1       (II)      .       2.04 Info
PLAT794_ALERT_5_G Tentative Bond Valency for Zn2       (II)      .       2.00 Info
PLAT860_ALERT_3_G Number of Least-Squares Restraints .............         50 Note
PLAT910_ALERT_3_G Missing # of FCF Reflection(s) Below Theta(Min).          2 Note
PLAT912_ALERT_4_G Missing # of FCF Reflections Above STh/L=  0.600        353 Note
PLAT915_ALERT_3_G No Flack x Check Done: Low Friedel Pair Coverage         79 %
PLAT915_ALERT_3_G No Flack x Check Done: Low Friedel Pair Coverage         79 %
PLAT933_ALERT_2_G Number of OMIT Records in Embedded .res File ...          1 Note
PLAT941_ALERT_3_G Average HKL Measurement Multiplicity ...........        4.9 Low
PLAT951_ALERT_5_G Calculated (ThMax) and CIF-Reported Kmax Differ           2 Units
PLAT961_ALERT_5_G Dataset Contains no Negative Intensities .......     Please Check
PLAT978_ALERT_2_G Number C-C Bonds with Positive Residual Density.          5 Info
```

---

0 **ALERT level A** = Most likely a serious problem - resolve or explain
0 **ALERT level B** = A potentially serious problem, consider carefully
6 **ALERT level C** = Check. Ensure it is not caused by an omission or oversight
21 **ALERT level G** = General information/check it is not something unexpected

0 ALERT type 1 CIF construction/syntax error, inconsistent or missing data
9 ALERT type 2 Indicator that the structure model may be wrong or deficient
5 ALERT type 3 Indicator that the structure quality may be low
8 ALERT type 4 Improvement, methodology, query or suggestion
5 ALERT type 5 Informative message, check

It is advisable to attempt to resolve as many as possible of the alerts in all categories. Often the minor alerts point to easily fixed oversights, errors and omissions in your CIF or refinement strategy, so attention to these fine details can be worthwhile. In order to resolve some of the more serious problems it may be necessary to carry out additional measurements or structure refinements. However, the purpose of your study may justify the reported deviations and the more serious of these should normally be commented upon in the discussion or experimental section of a paper or in the "special_details" fields of the CIF. checkCIF was carefully designed to identify outliers and unusual parameters, but every test has its limitations and alerts that are not important in a particular case may appear. Conversely, the absence of alerts does not guarantee there are no aspects of the results needing attention. It is up to the individual to critically assess their own results and, if necessary, seek expert advice.

Publication of your CIF in IUCr journals

A basic structural check has been run on your CIF. These basic checks will be run on all CIFs submitted for publication in IUCr journals (*Acta Crystallographica*, *Journal of Applied Crystallography*, *Journal of Synchrotron Radiation*); however, if you intend to submit to *Acta Crystallographica Section C* or *E* or *IUCrData*, you should make sure that full publication checks are run on the final version of your CIF prior to submission.

Publication of your CIF in other journals

Please refer to the *Notes for Authors* of the relevant journal for any special instructions relating to CIF submission.

PLATON version of 16/07/2020; check.def file version of 12/07/2020

Datablock ZnSedcdabco - ellipsoid plot

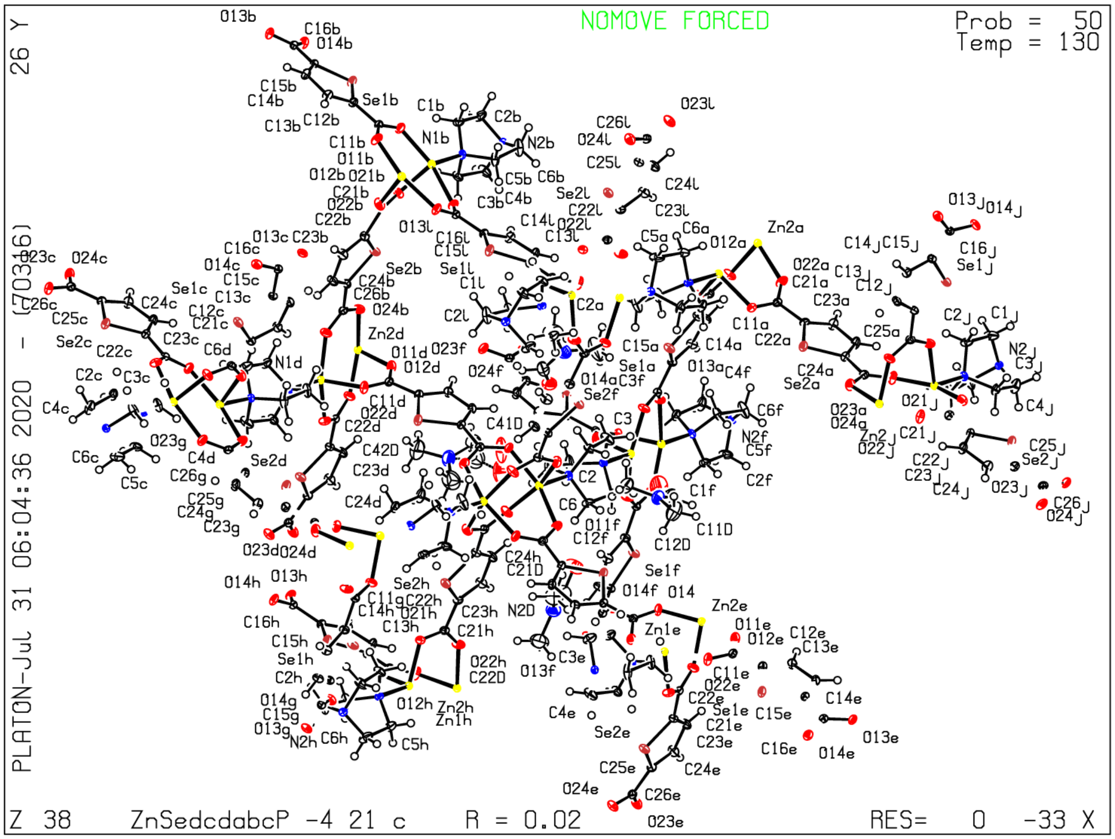

Supplement: Supplementary file 1 [file molecules-25-04396-s001.zip › checkcif.pdf]
