# Supplementary material for: A Selenophene-Incorporated Metal–Organic Framework for Enhanced CO2 Uptake and Adsorption Selectivity
Source: Molecules. 2020 Sep 24;25(19):4396. doi: 10.3390/molecules25194396 (PMC7582736; doi:10.3390/molecules25194396)
Supplement: Supplementary file 1 [file molecules-25-04396-s001.zip › Molecules_ESI.pdf]

Supporting Information

A Selenium Functionalized Metal-Organic Framework for Enhanced CO_2 Volumetric Uptake and Sorption Selectivity

Pavel A. Demakov,[1,2] Sergey S. Volynkin,[1] Denis G. Samsonenko,[1,2] Vladimir P. Fedin,[1,2] Danil N. Dybtsev[1,2]

[1]Nikolaev Institute of Inorganic Chemistry, Siberian Branch of the Russian Academy of Sciences, Novosibirsk 630090, Russia

[2]Novosibirsk State University, 2 Pirogova St., Novosibirsk 630090, Russia

[*] Correspondence: demakov@niic.nsc.ru

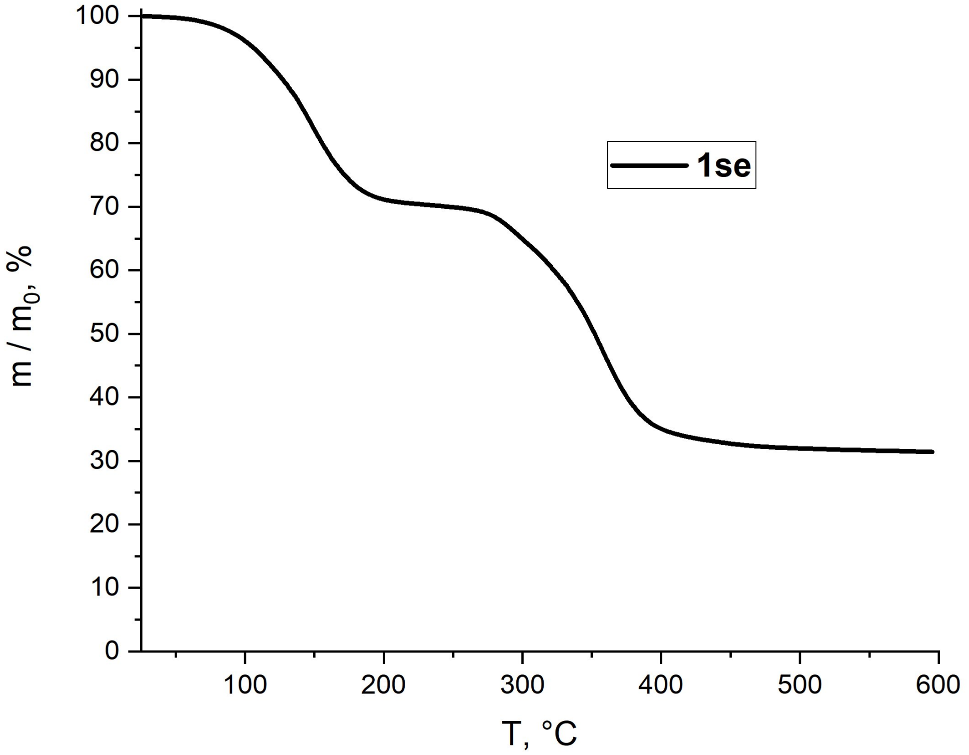

Figure S1. TG plot of the synthesized **1se** (black line).

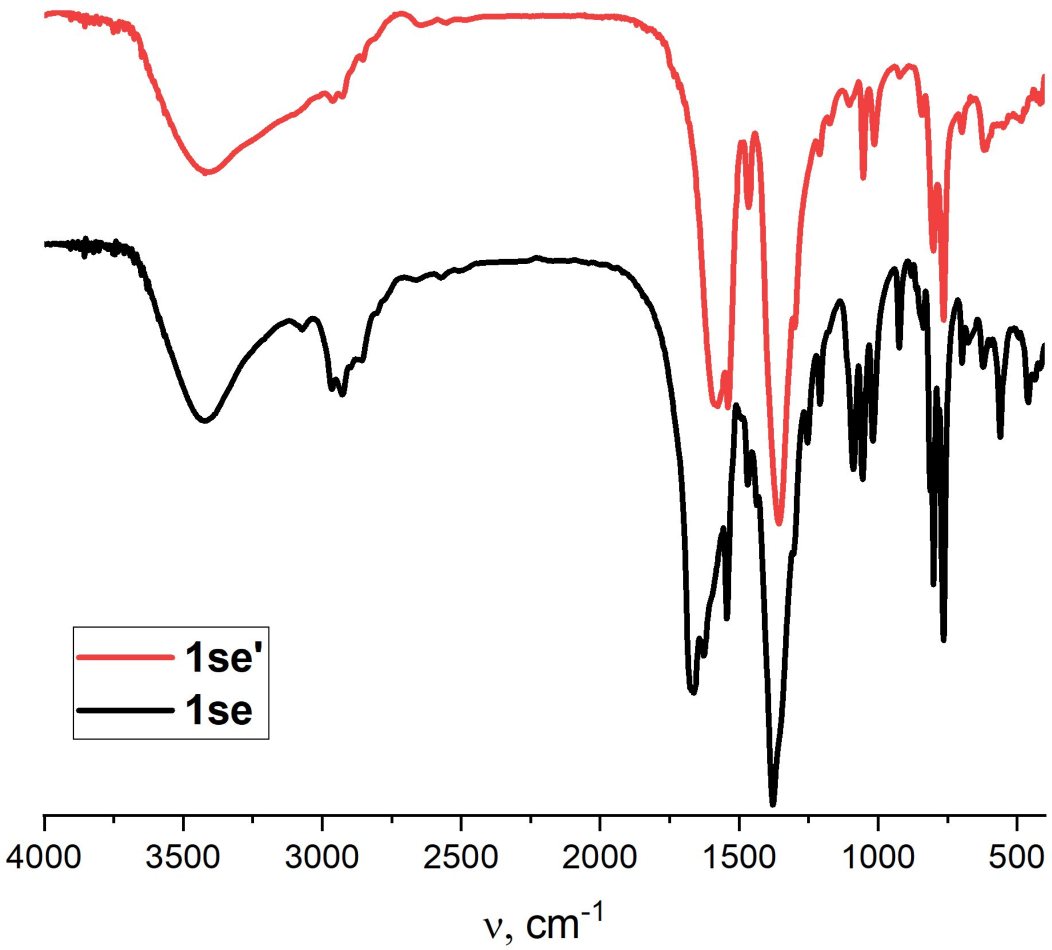

Figure S2. IR spectra of the synthesized **1se** (black line) and activated **1se′** (red line).

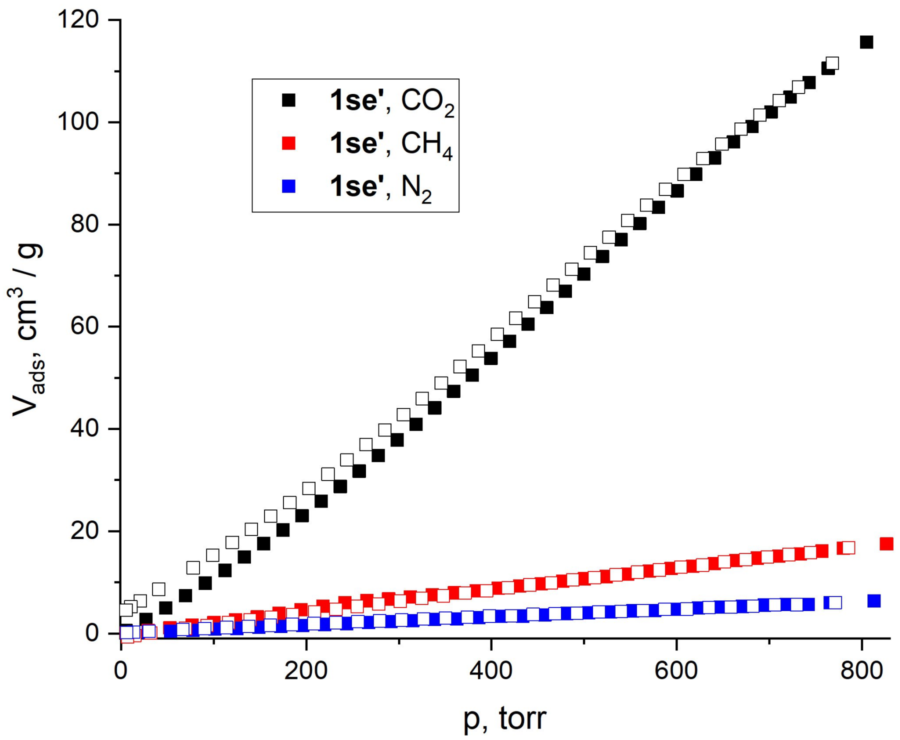

(a)

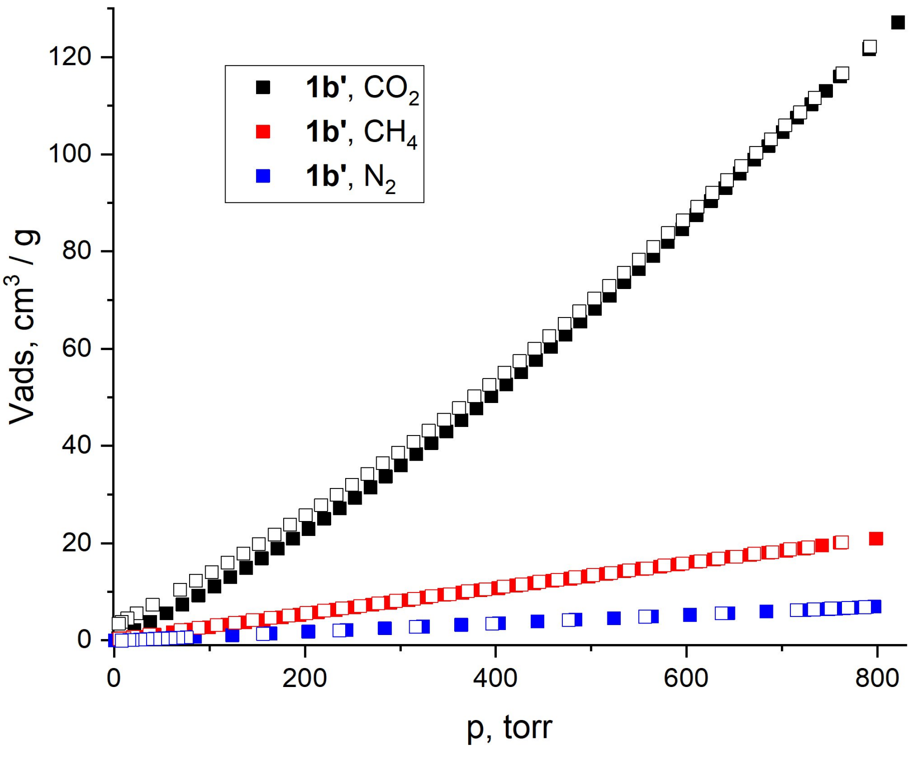

(b)

Figure S3. Gravimetric CO_2, CH_4 and N_2 adsorption and desorption isotherms for **1se′** (**a**) and **1b′** (**b**) at 273 K.

Table S1. Textural characteristics of **1se′**.

Model	A_{BET} ($m^2 \cdot g^{-1}$)		Model	V_{pore} ($cm^3 \cdot g^{-1}$)
Langmuir	1570		Gourvich<br>N_2 adsorption at<br>$p/p_0 = 0.95$	0.57
BET	1504			
DFT	1352		DFT model	0.42

Table S2. The comparison of porosities between the isoreticular **1t′**, **1se′** and **1b′**.

Compound	aperture, $Å^2$ (from the X-ray data)	V_{pore}, $cm^3 \cdot g^{-1}$ (from the X-ray data)	V_{pore}, $cm^3 \cdot cm^{-3}$ (experimental)
**1t′** [from Ref. 30]	5 x 8	0.68	0.64
**1se′**	5 x 8	0.55	0.60
**1b′**	6 x 8	0.75	0.62

Table S3. Calculated virial coefficients for CO_2 isosteric heats of adsorption and virial Henry constants determination**.**

MOF	Virial coefficients for CO_2 (Standard Error)			
	A_0	A_1	A_2	B_0
**1se′**	-2391.36 (0.29 %)	-43720.52 (1.5 %)	4892414 (2.7 %)	14.45192 (0.16 %)
**1b′**	-2290,35 (1.1 %)	-24036,42 (3.0 %)	-	14,084 (0.61 %)

Table S4. Fit parameters of the isotherms for IAST calculations.

Gas	Parameters (Standard Error)	
	**1se′**	**1b′**
CO_2	(273K) Freundlich isotherm<br>o = 0,00484 (0.31 %)<br>t = 0,88159 (0.70 %)	(273K) Freundlich isotherm<br>o = 0,00535 (0.19 %)<br>t = 0,80858 (0.60 %)
	(298 K) Freundlich isotherm<br>o = 0,00199 (0.15 %)<br>t = 0,89456 (0.34 %)	(298 K) Freundlich isotherm<br>o = 0,00203 (0.23 %)<br>t = 0,91471 (0.56 %)
CH_4	(273K) Henry isotherm<br>k = 0,0007040 (0.11 %)	(273K) Henry isotherm<br>k = 0,0008734 (0.13 %)
N_2	(273K) Henry isotherm<br>k = 0,0002607 (0.24 %)	(273K) Henry isotherm<br>k = 0,0002862(0.15 %)

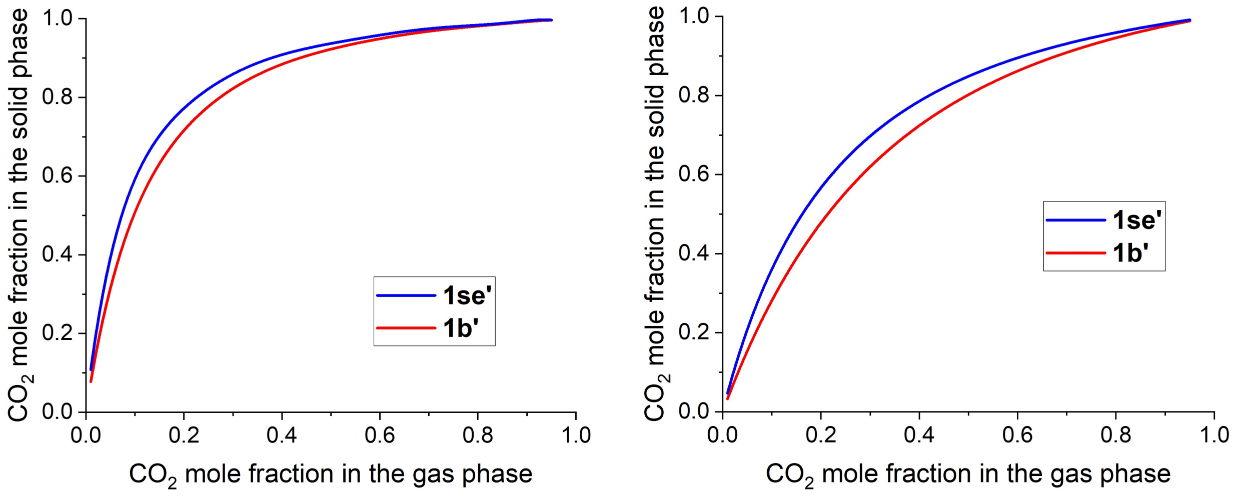

(a) **(b)**

Figure S4. Dependences of adsorbed CO_2 mole fraction in MOF on CO_2 mole fraction in gas phase for binary gas mixtures: CO_2/N_2 (**a**) and CO_2/CH_4 (**b**) at 273K and total P = 1 bar.

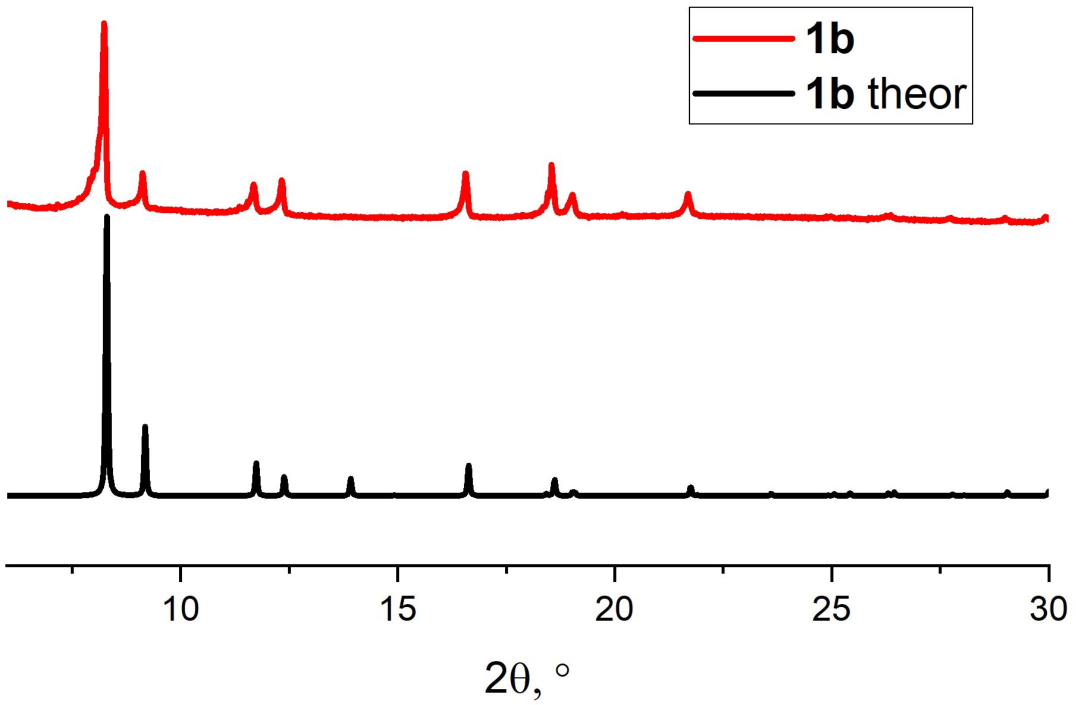

Figure S5. PXRD pattern of the synthesized compound **1b** (red line) compared to the theoretical one (black line).

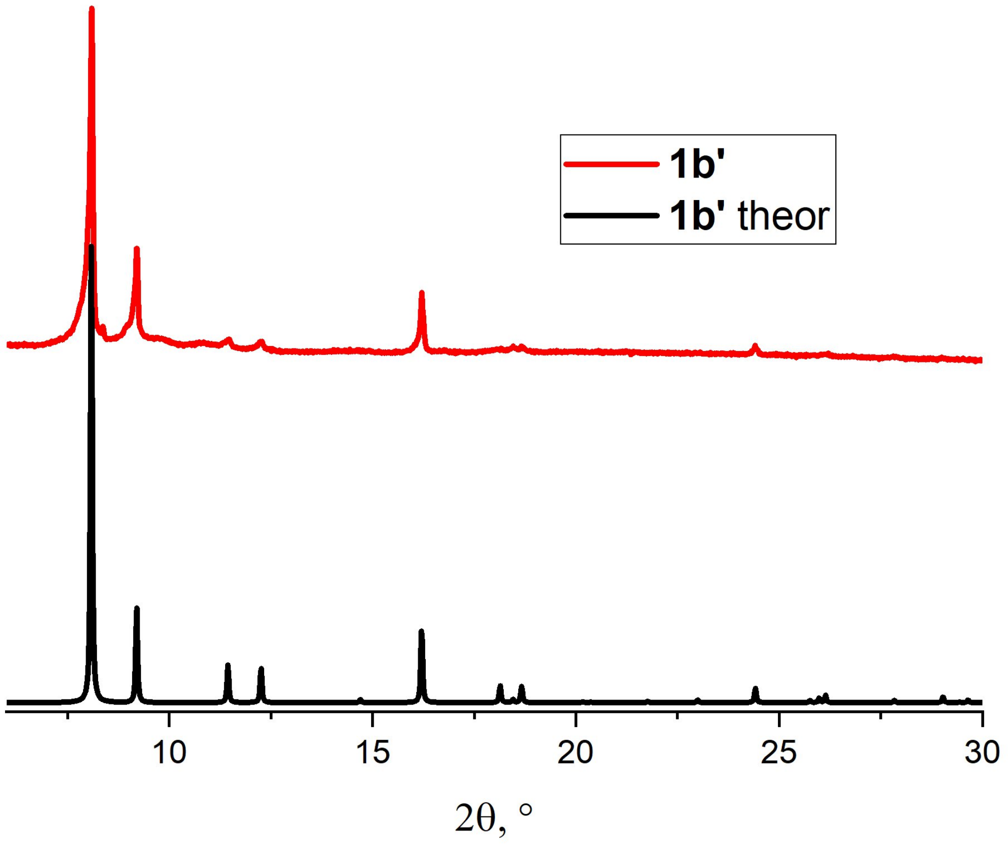

Figure S6. PXRD pattern of the synthesized compound **1b′** (red line) compared to the theoretical one for **1b′** (black line).
